# Functional characterization of *MLH1* missense variants unveils mechanisms of pathogenicity and clarifies role in cancer

**DOI:** 10.1371/journal.pone.0278283

**Published:** 2022-12-01

**Authors:** Marwa Mahdouani, Slim Ben Ahmed, Fahmi Hmila, Henda Rais, Rihab Ben Sghaier, Hanene Saad, Mariem Ben Said, Saber Masmoudi, Dorra Hmida, Angela Brieger, Stefan Zeuzem, Ali Saad, Moez Gribaa, Guido Plotz

**Affiliations:** 1 Laboratory of Human Cytogenetics, Molecular Genetics and Reproductive Biology, Farhat Hached University Hospital, Sousse, Tunisia; 2 Higher Institute of Biotechnology of Monastir, University of Monastir, Monastir, Tunisia; 3 Department of Oncology, Farhat Hached University Hospital, Sousse, Tunisia; 4 Faculty of Medicine Ibn El Jazzar of Sousse, University of Sousse, Sousse, Tunisia; 5 Department of General and Digestive Surgery, Farhat Hached University Hospital, Sousse, Tunisia; 6 Medical Service, Salah Azaiez Institute, Tunis, Tunisia; 7 Laboratory of Molecular and Cellular Screening Processes, Centre of Biotechnology of Sfax, Sfax, Tunisia; 8 Biomedical Research Laboratory, Department of Internal Medicine 1, University Hospital, Goethe University, Frankfurt am Main, Germany; University of Campania Luigi Vanvitelli: Universita degli Studi della Campania Luigi Vanvitelli, ITALY

## Abstract

Lynch syndrome is a heritable condition caused by a heterozygous germline inactivating mutation of the DNA mismatch repair (MMR) genes, most commonly the *MLH1* gene. However, one third of the identified alterations are missense variants, for which the clinical significance is unclear in many cases. We have identified three MLH1 missense alterations (p.(Glu736Lys), p.(Pro640Thr) and p.(Leu73Pro)) in six individuals from large Tunisian families. For none of these alterations, a classification of pathogenicity was available, consequently diagnosis, predictive testing and targeted surveillance in affected families was impossible. We therefore performed functional laboratory testing using a system testing stability as well as catalytic activity that includes clinically validated reference variants. Both p.(Leu73Pro) and p.(Pro640Thr) were found to be non-functional due to severe defects in protein stability and catalytic activity. In contrast, p.(Glu736Lys) was comparable to the wildtype protein and therefore considered a neutral substitution. Analysis of residue conservation and of the structural roles of the substituted residues corroborated these findings. In conjunction with the available clinical data, two variants fulfil classification criteria for class 4 “likely pathogenic”. The findings of this work clarify the mechanism of pathogenicity of two unclear *MLH1* variants and enables predictive testing and targeted surveillance in members of carrier families worldwide.

## Introduction

Lynch syndrome (LS) (MIM #120435) is an autosomal dominant disease characterized by increased lifetime risk and early onset of colorectal cancer (CRC) (70–80%), and increased incidence of other, extracolonic cancers such as endometrial cancer (50–60%), stomach cancer (13–19%), ovarian cancer (9–14%), cancer of the small intestine, biliary tract, brain, and carcinoma of the ureters and renal pelvis [1, 2]. It is a heritable condition caused by an heterozygous germline inactivating mutation of a DNA mismatch repair (MMR) gene, either *MLH1* (MIM# 120436), *MSH2* (MIM #609309), *MSH6* (MIM #600678), or *PMS2* (MIM #600259), or by deletions of the *EPCAM* gene’s 3’ region [3, 4]. Lynch syndrome patients require identification and subsequent inclusion in cancer detection and prevention schemes [5, 6].

Due to the lack of nationwide screening programs, Lynch syndrome and its associated cancer diseases are most likely under-diagnosed in Tunisia, therefore little is known about prevalence and the underlying genetics [7, 8]. However, age at cancer diagnosis in Tunisia is relatively low in a significant proportion of cases (14.2% of patients are < 40 years) [7], suggesting a significant contribution of genetic factors, as has been observed in other countries [9].

LS tumors are characterized by microsatellite instability (MSI), which results from somatic loss of the remaining wild-type allele of the affected MMR gene [10, 11]. However, the MSI phenotype is also observed due to somatic mutations in MMR genes or based on hypermethylation of the *MLH1* promoter in approximately 15% of sporadic colorectal cancers, therefore it is not a specific marker for LS [12–17]. *MLH1* promoter methylation is associated with a specific *BRAF* mutation (V600E) which is therefore used as an additional parameter for excluding patients from genetic testing since the tumor MSI in these cases is unlikely to result from a germline MMR gene inactivation [18, 19]. Promoter methylation can also be associated with genetic alterations and cause Lynch syndrome [20].

Since Lynch syndrome does not display a specific phenotypic expression allowing its diagnosis, combinations of clinical guidelines (Amsterdam criteria, Bethdesda guidelines) and the molecular tumor characteristics (MSI, IHC, BRAF mutation status) are applied to select patients for genetic analysis of mutations in MMR genes [21–24]. Subsequently, diagnosis is established if a causative (pathogenic) mutation in an MMR gene in the germline of the patient is found.

A significant proportion of germline variants are found in the *MLH1* gene, followed by the *MSH2* gene (50 and 40%, respectively), with only 10% found in the *MSH6* and *PMS2* genes [25]. The majority are missense variants with a high proportion whose clinical significance is unclear [26, 27]. These variants are referred to as variants of unclear significance (VUS) or unclassified variants (UV) which require pathogenicity classification [28]. As a result, diagnosis cannot be established, relatives of the patients are unable to receive predictive testing, and preventive surveillance cannot be properly targeted. It is therefore specifically important to interpret which small coding variants in the MMR genes are causative (pathogenic) and which represent neutral alterations.

While a plethora of *in silico* prediction algorithms of variant impact are available and useful for assessment of great numbers of uncharacterized variants [29], clinical decision making is still based on additional markers of pathogenicity or neutrality. The International Society for Gastrointestinal Hereditary Tumours (InSiGHT) has developed criteria for the interpretation of MMR gene variants. A systematic clinical classification of all variants contained in InSiGHT databases (http://insight-group.org/variants/database/) into a 5-tier-system was performed, based on a multifactorial bayesian quantitative approach or criteria combining qualitative clinical, population and functional data [30]. The bayesian quantitative approach starts using a *prior probability* of the variants’ pathogenicity calculated using an *in silico* algorithm [31], which is subsequently modified by clinical, diagnostic and functional data [32].

The 5-tiered-classification system represents the best, evidence-based approach for judging the clinical significance of unclear variants [33]. However, for many variants, no conclusive classification could yet be achieved due to insufficient information, hence they either remain un-classified or are attributed to class 3 (unclear). Consequently, continuing investigations into clinical and functional data of unclear variants and their carriers is mandatory.

This is the first time that *MLH1* functional analysis is performed for improving Lynch syndrome diagnosis in Tunisia. We have analyzed, for this purpose, three *MLH1* missense variations identified in Tunisian colorectal cancer patients. We show that the results of these functional analyses are consistent with information gained by analyzing residue conservation and protein structure. In conjunction with the clinical data provided for the Tunisian carrier patients, this information enables classification of two variants in class 4 (likely pathogenic). This allows a diagnosis to be established for carriers of these variants in Tunisia and elsewhere, and enables predictive testing for family members as well as better targeted surveillance measures and life style counselling [34].

## Materials and methods

### Patients

This study includes unpublished and published data from 6 Tunisian patients with suspected Lynch syndrome, including results from germline DNA sequencing, tumor testing (based on microsatellite instability analysis and immunohistochemistry), and family history [35]. For performing the genetic analyses, blood samples were obtained from the individuals and peripheral blood mononuclear cells (PBMCs) were separated for isolation of genomic DNA using standard procedures. Genetic and tumor analyses were performed as described before [35]. Samples and clinical data were anonymized prior to analysis.

The Bethesda II guidelines criteria were met by all families [22, 36]. The study was conducted in accordance with the Declaration of Helsinki, and approved by the Ethics and Research committee Farhat Hached University Hospital, Sousse, Tunisia, (OHRP IRB 00008931) on 10th May 2021. All patients were informed about their inclusion in the registries, and written informed consent was obtained from all participants during genetic counseling sessions including consent to use data in research. There were no deviations from the study protocol after approval was obtained.

### Sequencing, nomenclature and classification of genetic variants

The nomenclature guidelines of the Human Genome Variation Society (HGVS) were used to describe the detected genetic variants [37]. The nomenclature of all variants was checked using Mutalyzer [38]. The recurrence of the identified variants was established by interrogating four databases: the Leiden Open Variation Database (LOVD), ClinVar, Human Gene Mutation Database (HGMD). The InSiGHT database was used to check for current classifications of the variants [30].

### Cell lines

For this study, HEK293T cells were used. They were provided generously by Prof. Josef Jiricny, Zurich, Switzerland. Their identity was confirmed by comparing their genomic short tandem repeat (STR) profile from 9 loci to the source HEK293T cell line DSMZ ACC 635, and then by a variable number tandem repeats (VNTR) profile from the Leibnitz Institute DSMZ-German Collection of Microorganisms and Cell Cultures, Braunschweig, Germany, in 02/2009 and 06/2018. The cells used in this work were freshly thawed from frozen aliquots of these verified batches.

### Protein expression and quantification

The HEK293T cell line, pcDNA3-MLH1, and pSG5-PMS2 have been previously described [39]. Site-directed mutagenesis was used to generate missense variants using the Q5 Site directed mutagenesis system (New England Biolabs, Frankfurt, Germany) with appropriate primes according to the manufacturers’ protocols. All resulting plasmids were then confirmed by direct sequencing.

HEK293T cells in 10 cm round dishes were transiently transfected with 5 μg of vector DNA and 20 μL of polyethyleneimine (1 mg/mL, "Max" linear, 40 kDa, Polysciences, Warrington, PA) and extracted as previously described [40].

SDS-PAGE and immunoblotting were performed to examine the extracts using anti-MLH1, G168–728, BD Biosciences, and anti-PMS2, E-19, and anti-beta-Actin, C2 from Santa Cruz Biotechnologies. Chemiluminescence signals (Immobilon, Millipore) were detected and quantified using a Fuji LAS-4000 mini camera and Multi Gauge v3.2.

### Evaluation of expression defects with respect to pathogenicity

Protein expression and quantification were carried out in parallel with a stability-impaired neutral control variant (MLH1 p.(Val716Met)) and a severely destabilized pathogenic control variant (MLH1 p.(Ala681Thr)). A clinically pathogenic protein stability defect exists when the expression of the variant in question is similar to or lower than that of the pathogenic control variant [40–42].

### MMR activity

*In vitro* MMR activity of MLH1 variants was assessed using a validated procedure yielding clinically meaningful results [40, 43]. Briefly, protein extracts were mixed with 35 ng of DNA substrate containing a G-T mismatch and a 3’ single-strand nick at a distance of 83 bp. After 37°C incubation, the DNA substrate was purified and digested with EcoRV and AseI. The restriction fragments were separated in agarose gels and then analyzed with GelDoc XR plus detection and QuantityOne software (Bio-Rad). The repair efficiency (e) was calculated as follows: e = (intensity of repaired substrate bands)/ (intensity of all bands of substrate). This outcome is unaffected by the amount of DNA recovered during plasmid purification. Total repair efficiencies typically ranged between 50 and 90%. The repair efficiency of MLH1 variants was calculated as e (relative) = e (variant)/e (wild type) * 100 in comparison to a wild-type protein that had been produced in parallel.

### qPCR analysis of MLH1 transcription

As previously described [40] *MLH1* transcript levels were measured using quantitative PCR (qPCR) according to the MIQE guidelines [44]. Briefly, total RNA was extracted from transfected HEK293T cells using TRIzol (Invitrogen). cDNA was generated from 1 mg of total RNA, by reverse transcription using M-MLV reverse transcriptase (50U, RNase H Minus point mutant, Promega) and 250 ng of random primers (Promega) according to the manufacturers’ recommendations. Primer and probe sequences were designed using FileBuilder software and produced by Applied Biosystems.

Two reference genes were used *BLA* and *GAPDH* (assay #Hs99999905_m1, Applied Biosystems). The qPCR assays were conducted in a total volume of 15 mL, which included TaqMan universal master mix, an assay mixture containing the primers and hydrolysis probe, and 1.5 mL of a sample, with specific cycling conditions in a StepOnePlus Realtime cycler (Applied Biosystems). The StepOne 2.0 software was used to generate qPCR curves and Cq values.

To calculate *MLH1* transcript expression, the samples were normalized on the basis of the results for *GAPDH* [DCq = Cq(*MLH1*)-Cq(*GAPDH*)]. Subsequently, the variants were compared to the calibrator (wild-type *MLH1*) by calculating the DDCq value [DDCq(variant) = DCq(variant)-DCq(wild-type)]. Relative expression was calculated using the standard formula f = 2^-DDCq^.

### Structural and bioinformatic analyses

For assessment of residue conservation, an alignment containing >900 non-redundant full-length sequences of eukaryotic MLH1 proteins was generated using manually curated BLAST hits retrieved by interrogating the human MLH1 protein reference sequence NP_00240.1. Multiple hits from one organism were reduced to one, and non-MLH1 sequences were identified by virtue of the lacking highly conserved C-terminal FERC motif and also removed [45, 46].

Structural evaluations were conducted with an updated model of human MutLα (MLH1-PMS2) based on the structure of human PMS2-NTD [47] and homology models of MLH1- NTD and MLH1-PMS2-CTD [48–50]. Figures were generated using PyMOL v.1.4.1 (Schrodinger LLC). Presentations of sequence conservation were generated using WebLogo [51].

## Results

### Identification of three small coding *MLH1* variants and clinical features of carrier patients

**Fig 1:**
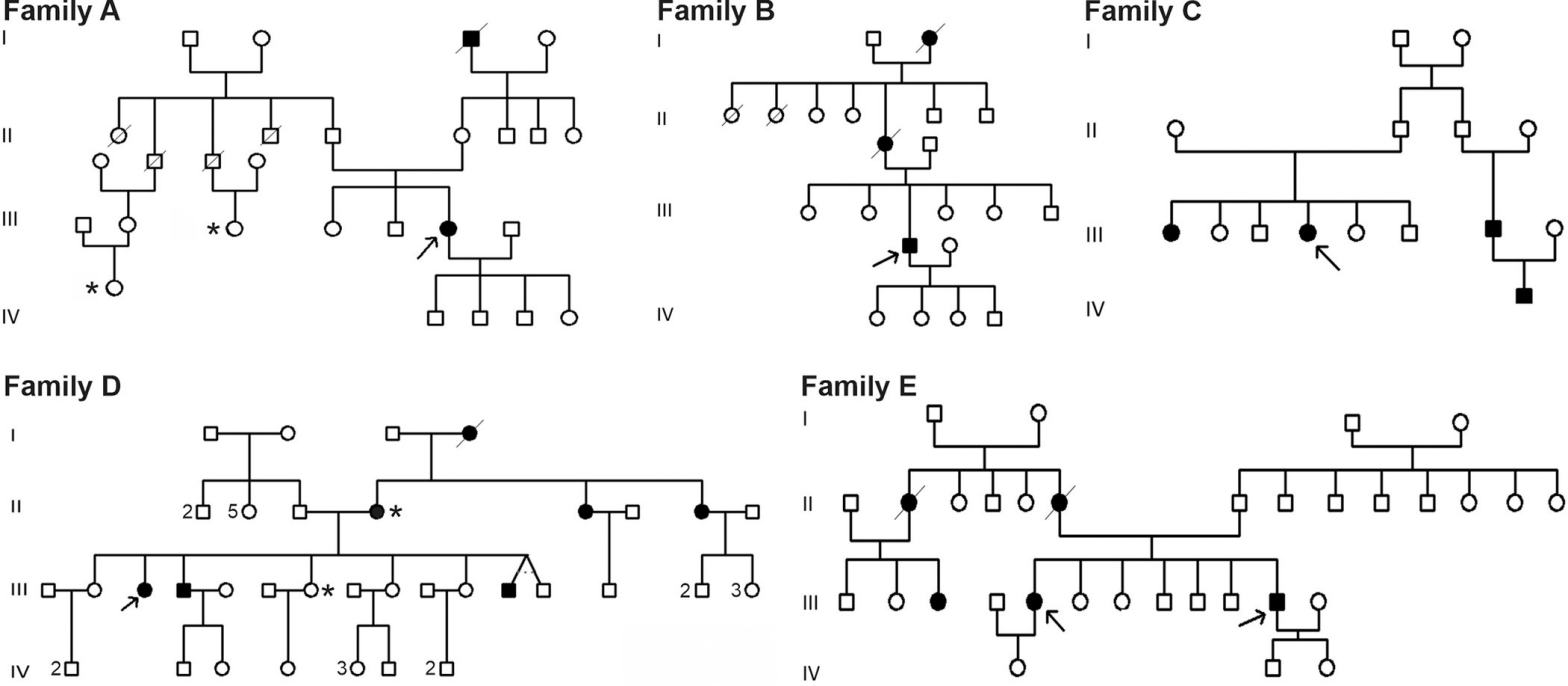
Pedigrees of *MLH1* variant carriers. Black filling of the symbols means patient with CRC. Numbers beside the symbols represent the number of sisters and brothers. Arrows indicate the index patients from Table 1. *breast cancer.

Three small coding variants were identified in six Tunisian patients meeting the Bethesda II guidelines (Table 1) [21]. For two variants (c.1918C>A, corresponding to p.(Pro640Thr), and c.2206G>A, corresponding to p.(Glu736Lys)), no classification of pathogenicity exists. The third variant (c.218T>C, corresponding to p.(Leu73Pro)), has been classified as VUS (class 3). Consequently, it is unclear if these three genetic variants are pathogenic, and if the carrier patients indeed have Lynch syndrome. For all affected patients, additional cancer cases in their families were present (Fig 1).

**Table 1:** Clinical and genetic information of the investigated Tunisian patients and functional variant evaluation.

Family	Pat-ient	Sex/Age at diagnosis	Tumor location	Results of immunohisto-chemistry	Alteration^1^ (SNP ID)	Alteration (protein)	Classifi-cation^2^/ MAPP PP2^3^	Functional evaluation (supporting evidence)
A	PIII.6	Female/52	Ascend. colon; Cecum	MSI-H Loss of MLH1 and PMS2	c.2206G>A	p.(Glu736Lys)	No classification/ 0.01	**Functional** (functionally neutral, unconserved)
B	PIII.3	Male/50	Right colon	-	c.1918C>A (rs63749792)	p.(Pro640Thr)	No classification/ 0.83	**Defective** (stability decreased like in pathogenic reference variant, highly conserved residue, consistent with role in structure, the similar substitution P640S is already classified “likely pathogenic”
C	PIII.4	Female/45	Transver. colon	MSI-H Loss of MLH1 and PMS2				
D	PIII.3	Female/46	Ascend. Colon	MSI-H Loss of MLH1 and PMS2				
E	PIII.11	Male/29	Left colon	MSI-H Loss of MLH1 and PMS2	c.218T>C (rs397514684)	p.(Leu73Pro)	class 3 (uncertain) (InSiGHT)/ 0.958	**Defective** (stability decreased like in pathogenic reference variant, no MMR activity, consistent with role in protein structure)
	PIII.5	Female/21	Cecum	-				

^1^gDNA alterations refer to transcript reference sequence NM_000249.3.

^2^Information on classifications according to InSiGHT consortium, retrieved in April 2022 from the LOVD database.

^3^MAPP PP2 prior probabilities of pathogenicity according to Thompson *et al*. [31].

The p.(Glu736Lys) variant occurred in one family (family A) with colon and breast cancers. DNA sequencing was carried out for one individual (PIII.6) who was diagnosed with colon cancer. His tumor showed loss of MLH1 and PMS2 proteins in IHC and displayed microsatellite instability (MSI-H). A right hemicolectomy was performed at the age of 52.

The variant p.(Pro640Thr) was found in a patient (PIII.3) of family B who had right-sided colon cancer. His mother (not tested) had been diagnosed with colon cancer at the age of 54 and died a few years later.

The same variant was discovered in two other intriguing families. In family C, this variant was found in one of the two sisters (Fig 1, (PIII.4)) from a consanguineous marriage diagnosed both with colon cancer at the age of 45. The index case whose CRC tumor showed MSI with loss of expression of MLH1 and PMS2 proteins, underwent total colectomy. Her sister was not tested and their parents were not diagnosed with colon cancer, but other members of their paternal family were (a cousin and his son).

From the eight members of family D, four brothers were diagnosed with colon cancer, all of whom underwent partial colectomy, including the index case (PIII.3) that was diagnosed at the age of 46 with tumor presenting loss of MLH1 and PMS2 proteins expression and MSI-H. One sister was diagnosed with breast cancer and underwent surgery. Their mother has colon and breast cancers, while her two sisters have colon cancer.

Finally, the p.(Leu73Pro) variant was discovered in two CRC patients in family E (PIII.11 and PIII.5). The CRC of patient PIII.11 showed an MSI phenotype and a loss of MLH1 and PMS2 protein expression in immunohistochemistry. He underwent a total colectomy. His sister (PIII.5) was diagnosed with cecum adenocarcinoma and underwent right hemicolectomy. Their mother died of colorectal cancer at the age of 50. Other family members on the maternal side were affected, but no one has been tested so far.

For none of the variants, allele frequencies of the variant allele have been reported in Tunisia or elsewhere, therefore they are not polymorphisms or frequent SNPs.

### Functional analysis by screening of the expression and MMR efficacy of MLH1 missense variants

In order to provide evidence if the identified alterations are causatively involved in the observed cancer cases, we performed functional analyses of these variants. Small coding variants frequently confer functional defects on the protein either by destabilization or by compromising its DNA repair activity [40, 52, 53]. We therefore assessed both functional parameters using a previously established and calibrated functional assay that evaluates both parameters [40, 43].

**Fig 2:**
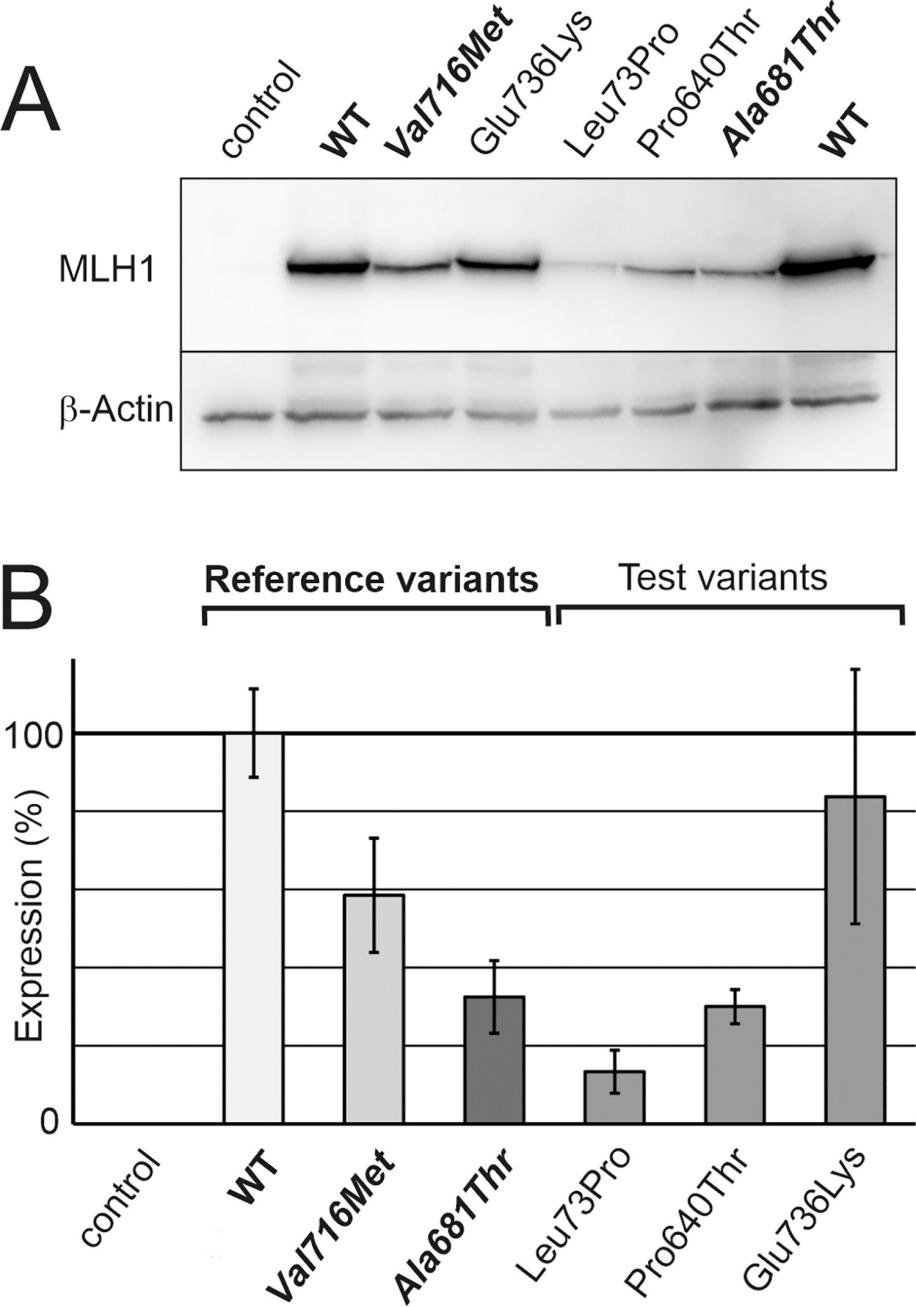
Analysis of expression of the MLH1 variants. A, Expression of wild-type and variant MLH1 proteins was visualized by SDS-PAGE and western blotting. The two stability reference variants p.(Ala681Thr) (pathogenic expression defect) and p.(Val716Met) (nonpathogenic expression defect, polymorphism) were transfected in parallel. The shown blots are representative for 7 independent experiments that were performed and delivered the data shown in evaluation (B). The shown blot is cropped, a full view of the blot is provided in the supplementary data. B, Average expression values in percent of the wild-type expression and standard deviations are shown for wild-type and variant MLH1 proteins.

The expression levels of the variants were assessed in direct comparison with the wild-type MLH1 protein and two previously established reference variants. MLH1 p.(Ala681Thr) serves to identify variants whose destabilization is so severe that it confers a pathogenic effect in humans due to low cellular protein levels. In contrast, the neutral polymorphism MLH1 p.(Val716Met) serves as a reference for clinically neutral stability defects [40]. Quantification of protein stability was performed by transient transfection in MLH1-deficient HEK293T cells, and resulting protein levels as assessed by SDS-PAGE and immunoblotting reflect protein stability (for representative blot, see Fig 2A) [40]. The average result of several independent experiments demonstrated that the p.(Glu736Lys) variant was similarly expressed as the wild-type protein and thus did not exhibit clinically relevant stability issues, as evident by comparison with expression of the p.(Val716Met) reference variant (Fig 2B). The p.(Pro640Thr) and p.(Leu73Pro) variants, on the other hand, strongly compromised stability, similarly strong as the reference variant for pathogenic stability defects (p.(Ala681Thr)). We performed quantification of the transcripts to confirm that mRNA expression was comparable (S1 Fig). This confirmed that the decrease in expression was not due to low mRNA expression after transient transfection, but reflected a destabilization of the variant protein, as demonstrated before [40].

In conclusion, there is insufficient MLH1 protein in the cell in case of the highly destabilized variants p.(Pro640Thr) and p.(Leu73Pro), therefore these variants confer a pathogenic defect due to protein destabilization. This is consistent with the absence of MLH1 in the immunohistochemical staining of the patients’ tumors (Table 1).

**Fig 3:**
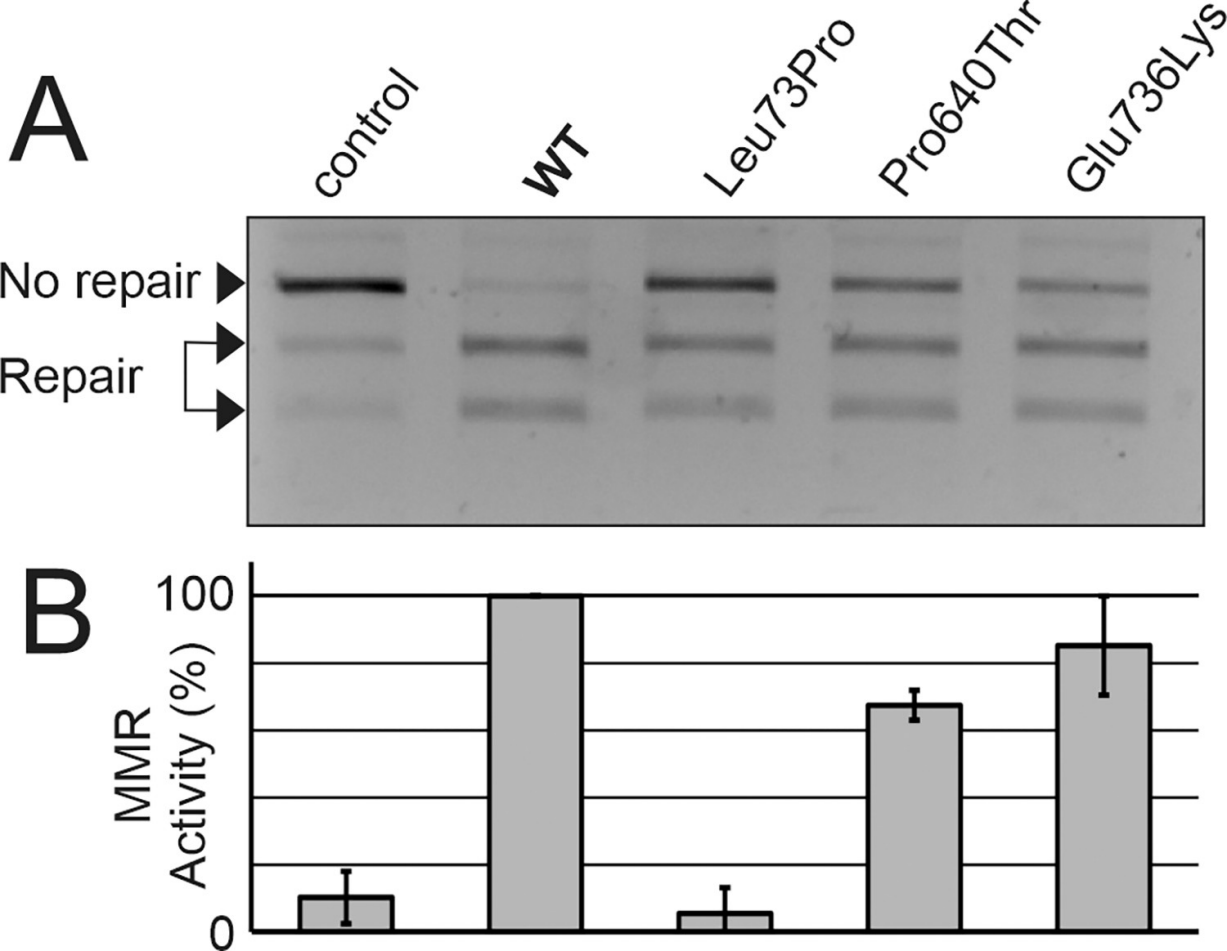
Analysis of mismatch repair activity of the MLH1 variants. DNA mismatch repair activity was assessed for wild-type and variant MLH1 proteins, and a negative control (without MLH1 protein) was included as detailed in “Materials and Methods”. **A**, Representative agarose gel image of the MMR activity measurement. The extent of repair is visible in the agarose gel electrophoresis by the generation of 2 smaller fragments (“Repair”) of the unrepaired, linearized plasmid (“No repair”). The shown agarose gel is cropped, a full view of the gel is provided in the supplementary data. **B**, 4 independent experiments were performed, and repair activity was scored relative to wild-type MLH1 protein (100%). Average repair values and standard deviations are shown.

The ability of the variants to perform the repair reaction *in vitro* was also assessed, as this is the main function of the MLH1 protein (Fig 3A).To validate the results, several independent experiments were carried out (Fig 3B).

The p.(Glu736Lys) and p.(Pro640Thr) variants showed MMR efficiency similar to that of the wild type (>60% of wild type activity), whereas repair activity of the p.(Leu73Pro) variant was similar to the negative control.

Taken together, functional assays at protein level revealed that p.(Leu73Pro) and p.(Pro640Thr) are variants that confer strong functional defects on the MLH1 protein. In contrast, the functionality of the p.(Glu736Lys) variant was indistinguishable from wildtype in the applied investigations.

### Conservation and structural roles of the affected residues

**Fig 4:**
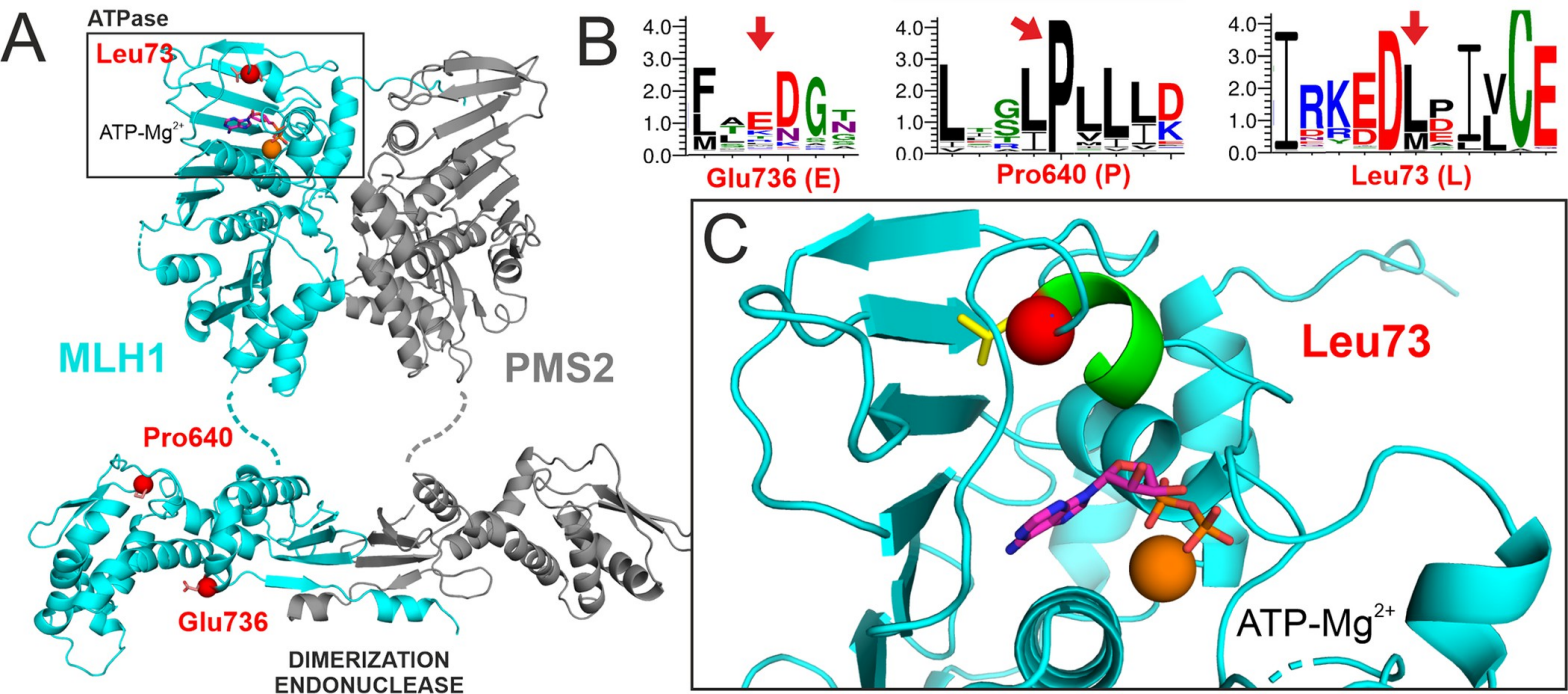
Structural positions of affected residues. A. The affected residues are shown as red balls in the structural model of the MLH1-PMS2 heterodimer (MLH1 shown in cyan, PMS2 in grey). The dimer comprises N-terminal ATPase domains (top, the bound ATP is shown in stick representation) in which the L73 residue is located. In the C-terminal domains, which confer dimerization and harbor the endonuclease function, the P640 and E736 residues are located. B. Weblogo presentation of sequence conservation of eukaryotic MLH1 in proximity of the three analyzed residues. These residues are marked by red arrows. Letter size corresponds to the degree of conservation. C. Detailed view of leucine 73 (red ball, side chain shown in yellow sticks) within the N-terminal ATPase pocket (area boxed in A). L73 is located within an α-helix (green) that contributes to formation of the nucleotide binding site. It engages in hydrophobic interactions with the extensive β-sheet forming the back of the ATPase domain.

Analysis of the structural positions and conservation of variant residues can explain and thereby confirm the results of functional studies. We therefore assessed the conservation and considered the positions of the affected residues within the structure of the MLH1-PMS2 heterodimer (Fig 4).

While both Glu736 and Pro640 are located in the C-terminal dimerization domain harboring the endonuclease function of MLH1, Leu73 is situated in the N-terminal ATPase domain (Fig 4A). The substitution of Glu736 to lysine did not cause a functional defect in our tests. The glutamic acid at position 736 is unconserved, and lysine (K) even naturally occurs in this position (Fig 4B). The residue is located far from the dimerization domain, and it side-chain is exposed to the solvent. Most likely, its role is providing a hydrophilic protein surface, and the actual charge of the side chain (positive or negative) does not play a relevant role. Taken together, there is no obvious reason why the conversion of Glu736 to the similarly hydrophilic residue lysine should impose constraints on structure or function, consistent with the functional measurements.

Pro640 is an extremely conserved internal residue within a highly hydrophobic loop comprising several conserved adjacent leucine residues (Fig 4B), suggesting that this proline is vital for formation of a hydrophobic core structure of the C-terminal domain. Any alterations disturbing the structural integrity of this hydrophobic pocket are likely to destabilize the protein, specifically since Pro640 is located within a protein region which we have found before to be prone to destabilization by substitutions [40]. Indeed, other substitutions of Pro640 have been observed in patients (Pro640Ser and Pro640Leu), and we have found a similar substitution to confer the same destabilizing effect (Pro640Ser) [40]. Consequently, the conversion of P640 to polar threonine residue is compatible with a disruption of protein structure and thus its stability, explaining the functional results we obtained.

Leu73 is located within an α-helix in the N-terminal ATPase pocket of MLH1 (Fig 4A). While it shows intermediate conservation, hydrophobic residues are highly conserved in this position (Fig 4B), which is consistent with this residue being involved in hydrophobic contacts within the interior of MLH1 (Fig 4C). Its substitution by proline can be expected to damage the α-helix conformation and distort the local structure of the ATPase pocket. This is compatible with the severe defect of stability observed for the p.(Leu73Pro) variant.

Taken together, both the information on residue conservation and the roles within protein structure are well in accordance with functional findings (Table 1).

## Discussion

In this study, we investigated three missense alterations in *MLH1* found in six Tunisian colorectal cancer patients from five families. Considering the sizes of the families of the index patients (Fig 1), many individuals in these families could benefit from predictive testing and targeted cancer surveillance. However, for neither of these variants a clinically applicable pathogenicity classification has been issued yet: Two alterations have not yet been addressed by the InSiGHT variant classification committee due to insufficient information, while one was provisionally classified as “uncertain” (class 3) due to insufficient or inconsistent information (Table 1).

If clinical information is insufficient for achieving a pathogenicity classification, functional data can provide additional evidence. However, functional data alone does not provide sufficient certainty for a pathogenicity classification either, therefore criteria require that functional data be combined with clinical observations. Moreover, it is relevant that the applied testing system for the functional data results in a reliable readout. For example, human test systems are preferable to those from other organisms to test variant gene and protein function, and those that provide a better characterized association of function with clinical effect are favorable. Several forms of functional test systems have been developed, based mostly on testing complementation of the variant proteins *in vitro* to MLH1-PMS2-deficient protein extracts and measuring the ability to perform a DNA MMR reaction [39, 52, 54, 55]. These *in vitro* systems test catalytic activity without specifically interrogating protein stability problems conferred by the alteration. While decreases in protein abundance also affect performance in MMR assay tests [56], it is not straightforward to translate (intermediate) functional defects into clinical outcomes.

In general, the majority of residues of any protein are involved in proper folding, structure and stability, while only a minor fraction are directly involved in catalysis. Consequently, the primary effect of the majority of missense alterations is the distortion of (local) folding, resulting in protein instability [57–59]. A catalytic deficiency may follow, either directly as a consequence of the folding problem, or indirectly, because the intracellular protein concentration sinks due to increased degradation of the mis-folded protein by the proteasome. Therefore, destabilization is an important parameter when assessing effects of human missense variations on health, and protein stability measurements are an important functional aspect assessed besides catalytic activity in other genetic diseases as well [60, 61].

When using stability measurements for pathogenicity assessment, a major question is which minimal protein concentration is required for normal cellular function, or to which extend of protein destabilization is tolerable. While MSH2 protein level reductions have been shown to be tolerated by MMR to as low as 10% [62], we have demonstrated that approximately 50% are required in case of MLH1 for MMR [40]. Moreover, our work allowed identification of an MLH1 stability reference variant p.(Ala681Thr) which is catalytically functional, but confers a defect in protein stability that is provably causative for Lynch syndrome [40]. For that reason, this variant is suitable for detecting pathogenic defects in stability by directly comparing expression levels of unclear *MLH1* variants with p.(Ala681Thr) [40–42].

We therefore apply an extended human test system that relies, besides determination of MMR activity, on clinically calibrated reference variants for detecting pathogenic versus innocuous stability defects (p.(Ala681Thr) and p.(Val716Met)) [40–43] (S1 Table). This procedure provides a benefit specifically in detecting pathogenicity caused by attenuated protein stability, which can evade detection in systems focusing on DNA mismatch repair activity. This can be exemplarily observed for the Lynch syndrome variant p.(Ala681Thr), whose defect is not detected in catalytic functional tests [55, 63]. The system is even capable of detecting pathogenic stability defects conferred by additive effects of two small coding variants [41]. Indeed, the majority of missense variants in MLH1 (and other disease-associated proteins) confer pathogenicity through protein destabilization [40, 52, 53, 57, 64, 65]. In previous analyses using the same methodology [40–42], we functionally analysed a total of 52 *MLH1* variants (S1 Table). Of these 52 variants, 29 (56%) were dysfunctional, with the vast majority (86%) showing a decrease of stability below the pathogenic reference stability limit provided by the p.(Ala681Thr) variant, while only 14% revealed their functional deficiency solely by loss of MMR activity.

24 of the 52 variants have in the meantime been classified either “pathogenic”/”likely pathogenic” or “not pathogenic”/”likely not pathogenic” (classes 4/5 or 1/2) by the InSiGHT classification committee using multiple lines of evidence. Importantly, all our functional results (100%) are consistent with the current InSiGHT classifications of these variants, confirming the reliability of the functional analysis.

Of those variants classified “pathogenic” or “likely pathogenic” (classes 4 or 5), 83% were dysfunctional due to insufficient protein stability in our measurement (lower than the pathogenic stability reference variant p.(Ala681Thr)). Consequently, this overview confirms our previous conclusion that stability determination provides a reliable and sensitive way of identifying pathogenicity in small coding MLH1 variants.

In terms of functional testing, this means that stability testing is a more direct and more sensitive way to identify the majority of functionally impaired variants. We have shown that expression levels after transient transfection correlate with variant protein stability and clinical outcome in patients [40].

Using these established procedures, we found that the MLH1 p.(Leu73Pro) variant displays a strong defect in stability and mismatch repair activity. This finding is consistent with the observation that the substitution is very likely to negatively affect the structural integrity of the ATPase pocket. The functional result is also congruent with previous evaluations on this variant [66]. Other substitutions of the same residue have been reported, but yeast assays have yielded inconclusive results for these [67]. In conclusion, the available evidence conclusively suggest that the variant is defective in function. For a formal pathogenicity classification to be applied in medical routine [30], an appropriate dysfunctional laboratory test can be used but must be complemented by sufficient clinical evidence [33]. Using the functional and clinical data provided in this study, this variant can be classified in class 4 (likely pathogenic) according to the InSiGHT classification rules (Table 2).

**Table 2:** Evaluation of the variants in terms of the InSiGHT variant classification criteria.

Criteria for class 4 (likely pathogenic)^1^	p.(Pro640Thr)	p.(Leu73Pro)
Variant-specific abrogated function in protein or mRNA based lab assay	++^2^	++^3^
*+ one of the following*		
• co-segregation with disease results in LR of >5:1	Insufficient data	Insufficient data
• ≥ 2 families with ≥ 2 affected non-prob and carriers	Insufficient data	Insufficient data
• ≥ 2 independent tumors with MSI and/or loss of MMR protein expression consistent with the variant location	+	+
	• family C (PIII.4)	• family E (PIII.5)
	• family D (PIII.3)	• family E (PIII.11)
		• carrier B1^3^

^1^ InSiGHT Variant Interpretation Committee: Mismatch Repair Gene Variant Classification

Criteria (Version 2.4 June 2018) for class 4 variants, abbreviated (for full text see https://www.insight-group.org/content/uploads/2018/08/2018-06_InSiGHT_VIC_v2.4.pdf)

^2^ this work and Takahashi *et al*. [53]

^3^ see also Borràs E., *et al*. [66]

The highly conserved residue Pro640 is a mutational hotspot located in an area in the C-terminus of MLH1 that is prone to destabilization by substitutions [40]. Correspondingly, the substitution to threonine caused a dramatic loss of stability, very comparable to the similar substitution p.Pro640Ser, which also renders MLH1 unstable [40] and which has already been classified “likely pathogenic” (class 4). The variant protein was largely active in MMR, consistent with a previous functional investigation [53], suggesting that the residue is not directly involved in catalytic activity of MLH1. However, our comparison with the clinically established stability reference variant p.(Ala681Thr) allowed to show that the stability defect of p.(Pro640Thr) is severe enough to cause a pathogenic functional defect. Taken together with the available clinical data, this variant also fulfils criteria for classification as “likely pathogenic” (class 4) (Table 2).

The finding that these two missense substitutions confer pathogenicity by protein destabilization is consistent with the abovementioned observation that this is the major path of pathogenicity in missense substitutions. Interestingly, this may also underlie the observation that penetrance in Lynch syndrome is comparable in carriers of *MLH1* missense alterations versus truncating alterations [68], since both essentially render the cell devoid of MLH1.

To our knowledge, this is the first report of the identification of the p.(Glu736Lys) variant. In the functional investigations, this variant retained a sufficiently high expression level that is above the clinical reference variant for proficient stability (p.(Val716Met)). These observations are consistent with the low conservation of the residue, and the evolutionary occurrence of lysine in this position, which contribute to a very low prior probability of pathogenicity (Table 1). Consequently, all available evidence suggests that this substitution is neutral.

In summary, we have performed for the first time functional testing for unclear MLH1 genetic variants identified in Tunisian cancer patients. In conjunction with clinical data of these patients, the functional results allow for classifying two of these variants into class 4 (likely pathogenic), while no functional defects could be found in another variant. These findings will improve genetic counselling and clinical management of affected Tunisian families and other carriers of these genetic variants worldwide.

## Supporting information

S1 Fig qPCR quantification of MLH1 cDNA after transfection. 48 h after transfection, cells were harvested and divided in two fractions, one for protein analysis and one for qPCR. qPCR was performed for quantification of MLH1 cDNA as detailed in Materials and Methods.
(TIF)

S1 Table Summary of previous functional measurements and current InSiGHT classifications. The table lists functional results of previously performed MLH1 variant measurements, sorted by stability (expression of WT in %). All variants with stability measurements lower than the clinical stability reference variant for pathogenicity (p.(A681T)) are indicated with red colour, those with expression higher than the neutral stability reference variant (p.(V716M)) are indicated by green colour. For MMR activity measurements, activity close to WT (>75%) was considered proficient, activity close to negative control (<25%) was considered deficient. Functional result summary can be compared to current variant classifications of the InSiGHT (retrieved October 2022).
(PDF)

S1 Raw images (PDF)
