# Supplementary figures and images for: Functional characterization of MLH1 missense variants unveils mechanisms of pathogenicity and clarifies role in cancer
Source: PLoS One. 2022 Dec 1;17(12):e0278283. doi: 10.1371/journal.pone.0278283 (PMC9714755; doi:10.1371/journal.pone.0278283)

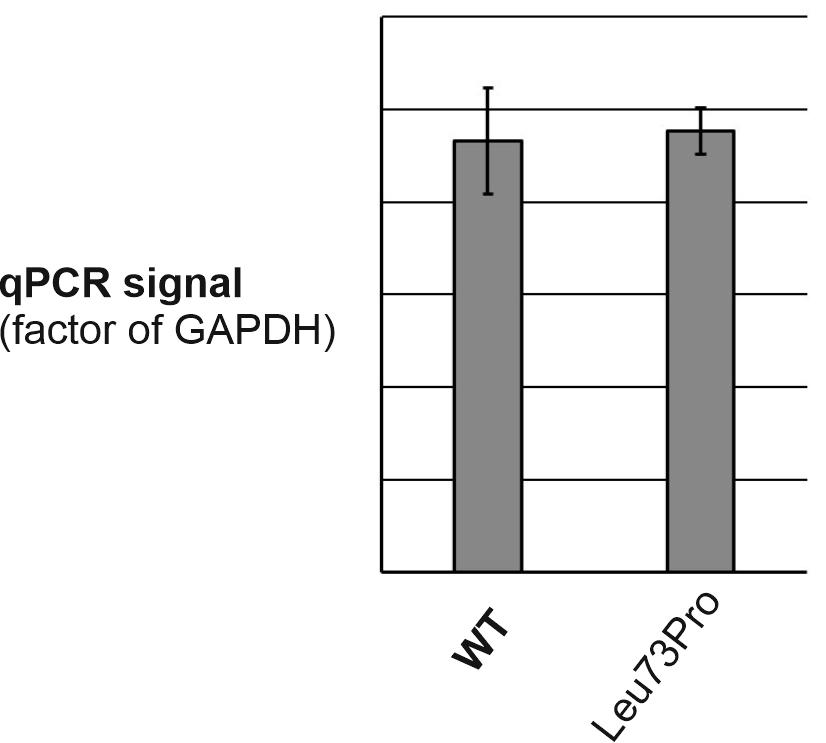

Supplement: S1 Fig — 48 h after transfection, cells were harvested and divided in two fractions, one for protein analysis and one for qPCR. qPCR was performed for quantification of MLH1 cDNA as detailed in Materials and Methods. (TIF) [file pone.0278283.s001.tif]
