# Supplementary material for: Functional characterization of MLH1 missense variants unveils mechanisms of pathogenicity and clarifies role in cancer
Source: PLoS One. 2022 Dec 1;17(12):e0278283. doi: 10.1371/journal.pone.0278283 (PMC9714755; doi:10.1371/journal.pone.0278283)
Supplement: S1 Table — The table lists functional results of previously performed MLH1 variant measurements, sorted by stability (expression of WT in %). All variants with stability measurements lower than the clinical stability reference variant for pathogenicity (p.(A681T)) are indicated with red colour, those with expression higher than the neutral stability reference variant (p.(V716M)) are indicated by green colour. For MMR activity measurements, activity close to WT (>75%) was considered proficient, activity close to negative control (<25%) was considered deficient. Functional result summary can be compared to current variant classifications of the InSiGHT (retrieved October 2022). (PDF) [file pone.0278283.s002.pdf]

S1 Table: Summary of previous functional measurements and current InSiGHT classifications

The table lists functional results of previously performed MLH1 variant measurements, sorted by stability (expression of WT in %). All variants with **stability measurements** lower than the clinical stability reference variant for pathogenicity p.(Ala681Thr) are indicated with red colour, those with expression higher than the neutral stability reference variant p.(V716M) are indicated by green colour. For **MMR activity** measurements, activity close to WT (>75%) was considered proficient, activity close to negative control (<25%) was considered deficient. Functional result summary can be compared to current variant classifications of the InSiGHT (retrieved October 2022).

Variant	Results functional analysis in Hinrichsen *et al.* (2013)[1]			Classification InSiGHT (10/2022)[2]	
	Expression % of WT<br>>V716M=proficient<br><A681T=deficient	MMR activity % of WT<br>>75=proficient<br><25=deficient	Functional result summary	Class	Class in words
**WT**	100	100	proficient (MMR and stability)		
**I219V**	94	97	proficient (MMR and stability)	1	not pathogenic
**R265H**	91	91	proficient (MMR and stability)	-	no classification
**L749P**	85	15	MMR deficient	5	pathogenic
**E663G**	85	95	proficient (MMR and stability)	-	no classification
**D601G**	82	96	proficient (MMR and stability)	3	uncertain
**K618A**	78	92	proficient (MMR and stability)	1	not pathogenic
**K84E**	76	9	MMR deficient	4	likely pathogenic
**H718Y**	73	110	proficient (MMR and stability)	1	not pathogenic
**R755W**	72	10	MMR deficient	3	uncertain
**E578G**	71	91	proficient (MMR and stability)	1	not pathogenic
**H109Q**	71	71	unclear (MMR)	3	uncertain
**V716M**	64	88	**STABILITY REFERENCE VARIANT 1[5]**	1	not pathogenic
**R522W**	62	105	unclear	-	no classification
**R659Q**	61	85	unclear	3	uncertain
**V506A**	58	88	unclear	3	uncertain
**N551T**	53	80	unclear	3	uncertain
**G54E**	52	44	unclear	-	no classification
**A681T**	52	99	**STABILITY REFERENCE VARIANT 2[5]**	5	pathogenic
**A586P**	50	21	Stability and MMR deficient	3	uncertain
**L559R**	45	79	Stability deficient	3	uncertain
**T117M**	44	5	Stability and MMR deficient	5	pathogenic
**L622H**	42	80	Stability deficient	5	pathogenic
**T82I**	41	6	Stability and MMR deficient	5	pathogenic
**P640S**	30	80	Stability deficient	4	likely pathogenic
**R659L**	28	8	Stability and MMR deficient	-	no classification
**L574P**	27	16	Stability and MMR deficient	4	likely pathogenic
**R725C**	26	101	Stability deficient	3	uncertain
**P654L**	25	67	Stability deficient	5	pathogenic
**L676R**	24	12	Stability and MMR deficient	3	uncertain
**P648L**	22	92	Stability deficient	4	likely pathogenic
**T662P**	22	93	Stability deficient	-	no classification
**D41G**	20	10	Stability and MMR deficient	5	pathogenic
**G65D**	17	19	Stability and MMR deficient	4	likely pathogenic
**G101D**	16	11	Stability and MMR deficient	4	likely pathogenic
**N38H**	14	15	Stability and MMR deficient	5	pathogenic
**R659P**	13	99	Stability deficient	5	pathogenic
**L653R**	11	5	Stability and MMR deficient	4	likely pathogenic
**E102K**	2	27	Stability deficient	-	no classification

Variant	Results functional analysis in Hinrichsen *et al.* (2014)[3]			Classification InSiGHT (10/2022)[2]	
	Expression % of WT<br>>V716M=proficient<br><A681T=deficient	MMR activity % of WT<br>>75=proficient<br><25=deficient	Functional result summary	Class	Class in words
**Q689R**	106	99	proficient (MMR and stability)	1	not pathogenic
**L507F**	101	91	proficient (MMR and stability)	-	no classification
**V716M**	83	-	**STABILITY REFERENCE VARIANT 1[5]**	1	not pathogenic
**D41H**	70	11	MMR deficient	5	pathogenic
**E605del**	70	89	unclear	-	no classification
**A681T**	52	-	**STABILITY REFERENCE VARIANT 2[5]**	5	pathogenic
**E605del+V716M**	44	87	Stability deficient	-	no classification

Variant	Results functional analysis in Köger *et al.* (2018)[4]			Classification InSiGHT (10/2022)[2]	
	Expression % of WT<br>>V716M=proficient<br><A681T=deficient	MMR activity % of WT<br>>75=proficient<br><25=deficient	Functional result summary	Class	Class in words
**N338S**	130	95	proficient (MMR and stability)	3	uncertain
**R575K**	121	101	proficient (MMR and stability)	-	no classification
**I500del**	105	104	proficient (MMR and stability)	-	no classification
**H112Q**	103	72	unclear (reduced MMR)	-	no classification
**P141A**	92	111	proficient (MMR and stability)	3	uncertain
**V716M**	84	-	**STABILITY REFERENCE VARIANT 1[5]**	1	not pathogenic
**Y97D**	81	27	unclear	-	no classification
**A681T**	61	-	**STABILITY REFERENCE VARIANT 2[5]**	5	pathogenic
**R265P**	60	29	Stability deficient	4	likely pathogenic
**L676P**	41	49	Stability deficient	3	uncertain
**K616del**	40	35	Stability deficient	-	no classification

[1] Hinrichsen I, Brieger A, Trojan J, Zeuzem S, Nilbert M, Plotz G. **Expression defect size among unclassified MLH1 variants determines pathogenicity in Lynch syndrome diagnosis**. *Clin Cancer Res*. 2013; 19:2432–41. doi: 10.1158/1078-0432.CCR-12-3299 PMID: 23403630.

[2] MLH1 variant pathogenicity classifications performed by experts according to current guidelines in 5-tiered system, retrieved from https://www.insight-database.org/classifications/ in October 2022.

[3] Hinrichsen I, Schäfer D, Langer D, Köger N, Wittmann M, Aretz S, et al. **Functional testing strategy for coding genetic variants of unclear significance in MLH1 in Lynch syndrome diagnosis**. *Carcinogenesis*. 2015; 36:202–11. doi: 10.1093/carcin/bgu239 PMID: 25477341.
[4] Köger N, Paulsen L, López-Kostner F, Della Valle A, Vaccaro CA, Palmero EI, et al. **Evaluation of MLH1 variants of unclear significance**. *Genes Chromosomes Cancer*. 2018; 57:350–8. doi: 10.1002/gcc.22536 PMID: 29520894.
[5] **MLH1 protein stability reference variants** were extensively established in reference [1]. **Stability referece variant 1** (**V716M=p.(Val716Met**)) represents a MLH1 polymorphism that displays reduced protein stability, but does not entail any effects on human health. It is therefore used to judge if a stability defect in an unclassified MLH1 variant is clinically neutral (when expression is higher than V716M). **Stability reference variant 2** (**A681T=p.(Ala681Thr)**) is a variant that displays WT MMR activity, but a protein stability stronger reduced than V716M. A681T is pathogenic, and carriers of the A681T variant have Lynch syndrome (although this variant has a lower penetrance). For these reasons, the variant is used as reference variant for protein stability defects that confer a pathogenic phenotype.
