# Supplementary material for: Functional characterization of MLH1 missense variants unveils mechanisms of pathogenicity and clarifies role in cancer
Source: PLoS One. 2022 Dec 1;17(12):e0278283. doi: 10.1371/journal.pone.0278283 (PMC9714755; doi:10.1371/journal.pone.0278283)

Raw image of Figure 2A-panel 1
(western blot detected with anti-MLH1)

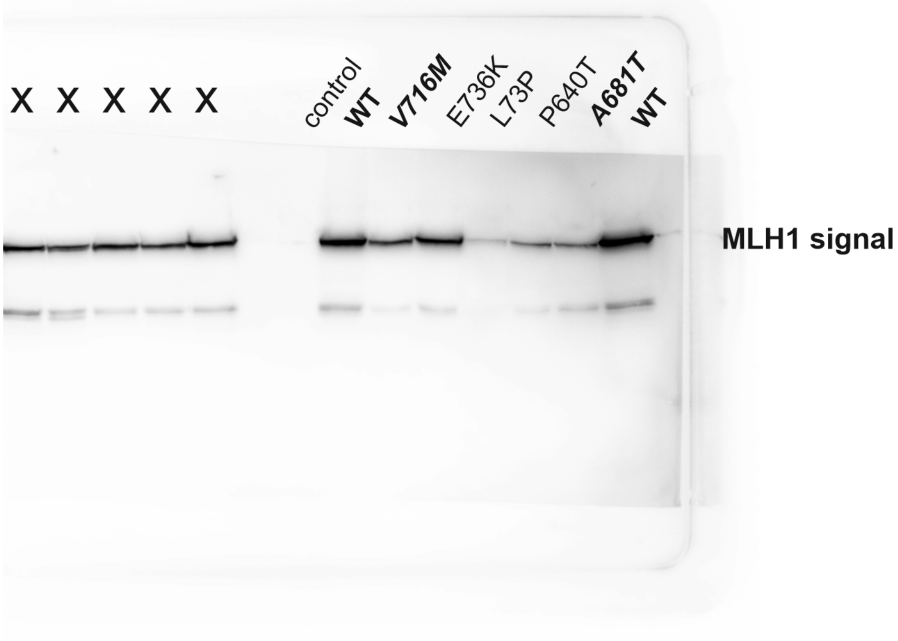

Raw image of Figure 2A-panel 2
(western blot detected with anti-MLH1, -PMS2, -b-Actin)

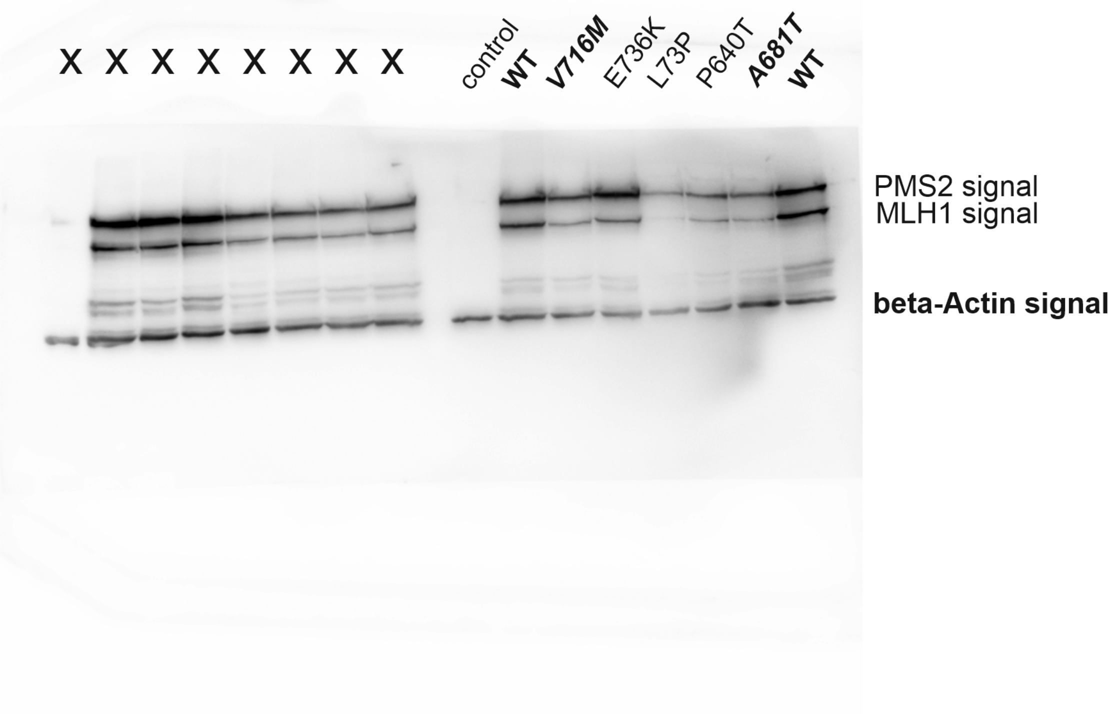

Raw image of Figure 3A (agarose gel)

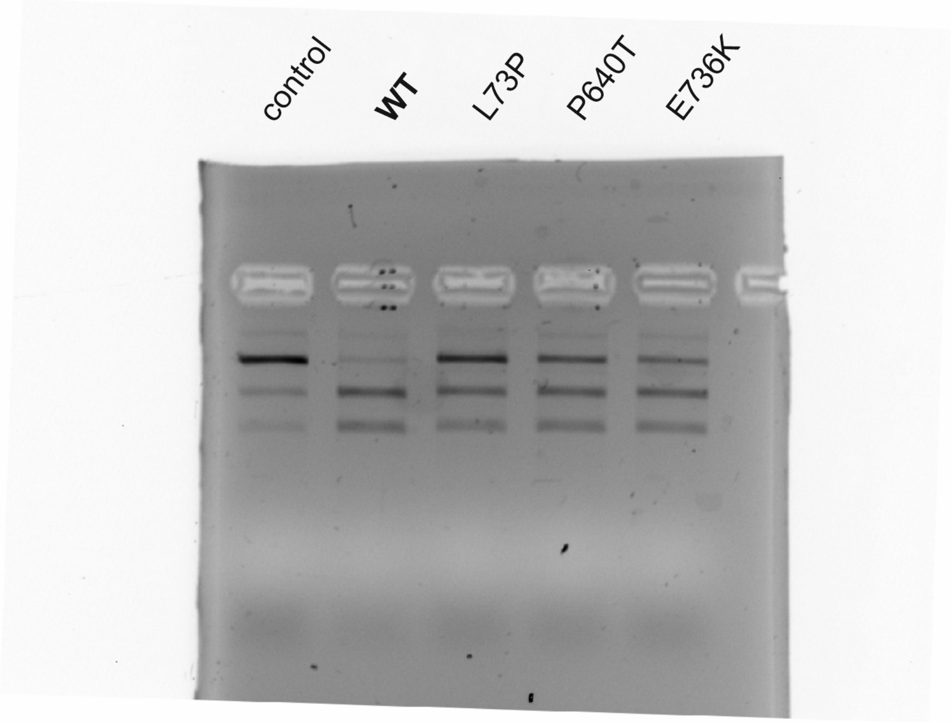

Supplement: S1 Raw images — (PDF) [file pone.0278283.s003.pdf]
